# Supplementary material for: Hybrid Models and Biological Model Reduction with PyDSTool
Source: PLoS Comput Biol. 2012 Aug 9;8(8):e1002628. doi: 10.1371/journal.pcbi.1002628 (PMC3415397; doi:10.1371/journal.pcbi.1002628)
Supplement: Text S4 — Complete source code for the PyDSTool package (version 0.88.120504). Includes API documentation and help files linking to web pages. This file is identical to the current public release on Sourceforge.net. (ZIP) [file pcbi.1002628.s004.zip › PyDSTool/html/PyDSTool.Toolbox.phaseplane.max_curvature_zone_leaf-class.html]

xml version="1.0" encoding="ascii"?


PyDSTool.Toolbox.phaseplane.max\_curvature\_zone\_leaf


| Home | Trees | Indices | Help | PyDSTool |
| --- | --- | --- | --- | --- |

Package PyDSTool ::
Package Toolbox ::
Module phaseplane ::
Class max\_curvature\_zone\_leaf

[hide private]

[frames] | no frames]

# Class max\_curvature\_zone\_leaf

source code

```
       object --+                    
                |                    
 MProject.feature --+                
                    |                
MProject.feature_leaf --+            
                        |            
 MProject.ql_feature_leaf --+        
                            |        
                    zone_leaf --+    
                                |    
              nullcline_zone_leaf --+
                                    |
                                   max_curvature_zone_leaf
```

---

```
A single zone of locally maximal curvature

pars:
  xtol --> precision of zone center finding
  direction (currently ignored -- looks for both)
  min_curvature_pc --> percentage of the maximum curvature found at sample
     points for which a local minimum value of curvature must meet to be
     considered a zone.
  find_exact -> exact x value of max curvature (not implemented yet)
  zone_width -> positive scalar (default Inf grows to next local maximum)
```


|  |  |
| --- | --- |
| Instance Methods | [hide private] |

|  |  |
| --- | --- |
| evaluate(self, nullc) | source code |

**Inherited from `nullcline_zone_leaf`** (private):
`_grow_zone_by_radius`,
`_grow_zone_by_width`,
`_prepare`,
`_set_mutex_zone_by_width`

**Inherited from `zone_leaf`**:
`finish`

**Inherited from `MProject.feature_leaf`**:
`__repr__`,
`__str__`,
`set_ref_traj`

**Inherited from `MProject.feature_leaf`** (private):
`_residual_info`

**Inherited from `MProject.feature`**:
`__call__`,
`__eq__`,
`__hash__`,
`__init__`,
`__ne__`,
`info`,
`postprocess_ref_traj`,
`prepare`,
`reset_metric`,
`validate`

**Inherited from `MProject.feature`** (private):
`_find_idx`

**Inherited from `object`**:
`__delattr__`,
`__getattribute__`,
`__new__`,
`__reduce__`,
`__reduce_ex__`,
`__setattr__`


|  |  |
| --- | --- |
| Properties | [hide private] |

**Inherited from `object`**:
`__class__`


|  |  |
| --- | --- |
| Method Details | [hide private] |

|  |  |
| --- | --- |
| evaluate(self, nullc) | source code |

Overrides: MProject.feature.evaluate

  


| Home | Trees | Indices | Help | PyDSTool |
| --- | --- | --- | --- | --- |

|  |  |
| --- | --- |
| Generated by Epydoc 3.0.1 on Fri May 4 15:24:09 2012 | http://epydoc.sourceforge.net |
